# Does Vitamin Supplementation Play a Role in Chronic Kidney Disease?

**DOI:** 10.3390/nu15132847

**Published:** 2023-06-23

**Authors:** Aleksandra Beata Juszczak, Maciej Kupczak, Tomasz Konecki

**Affiliations:** 1st Department of Urology, Medical University of Lodz, 90-549 Lodz, Polandtomasz.konecki@umed.lodz.pl (T.K.)

**Keywords:** chronic kidney disease, supplementation, vitamins, dialysis

## Abstract

Although the role of vitamins in the human body is proven, guidelines for patients with chronic kidney disease (CKD) remain unclear. This narrative review summarizes the findings of 98 studies of CKD and the effects of vitamin D, B, C, A, E, and K supplementation on patients on dialysis for CKD, with the aim of summarizing the existing guidelines. The findings are promising, showing the potential effectiveness of vitamin supplementation with, for example, vitamins B, D, or C. However, recommendations are still ambiguous, especially in the case of vitamins A and K, due to the potential toxicity associated with higher doses for patients. Continued research is needed to rigorously evaluate the effectiveness and carefully consider the potential risks of some vitamin supplementation for patients with CKD.

## 1. Introduction

Due to increasing life expectancy and the significant development of society, there has been a notable rise in the prevalence of civilization diseases worldwide. Chronic kidney disease (CKD) is considered one of them. It is usually associated with obesity, hypertension, and diabetes [1,2]. Body fat, muscle mass, and overall body mass index (BMI) have been proven to be independent and directly associated with survival in patients with CKD [2,3]. The scientific community currently focuses on preventive action to stop the development of the disease and reduce its impact on overall health, or at least inhibit its development [4,5]. The treatment of CKD is based on inhibiting kidney damage, preventing malnutrition, and reducing the severity of metabolic disorders [6]. Various studies suggest that a reduced nutritional status may be both a predictor and a cause of death in people with CKD [2,7,8,9]. Many studies have focused on the contribution of proteins, fats, and macrominerals to the diet of people with chronic kidney disease [10]. Over the past few years, several studies have been published on the vitamin requirements of patients with CKD [7,11,12]. It is believed that some drugs may interfere with the metabolism or action of certain vitamins, including vitamin B6, folic acid, and riboflavin [7,12]. Comorbidities can contribute to low intake, digestive disorders, absorption, or the effects of certain vitamins, and they may also require drugs that interfere with the effects of vitamins [7,11,13]. Because the role of vitamins in the human body is considerable, research has been focused on the role of diet and supplementation in chronic kidney disease [5,7,13,14].

This article aims to present a narrative review of the effect of vitamin supplementation with vitamins such as D, B, C, A, E, and K on patients on dialysis due to chronic kidney disease.

## 2. Chronic Kidney Disease

Chronic kidney disease (CKD) is a significant public health problem worldwide [15,16]. Out of the world population, the prevalence of CKD, with the majority being stage 3, is between 11 and 13% [17]. Although most patients with CKD can generally be classified as having mild disease, which is represented by stages 1 and 2, a significant number present moderate to severe disease, classified as stages 3 and 4. The total number of patients with stage 5 CKD (also referred to as end-stage renal disease (ESRD)) is steadily increasing [1,10]. The progressive deterioration of kidney function increases the production of oxidative stress mediators [4,15]. The slow, irreversible, and progressive deterioration of kidney function, characterized by a decrease in the glomerular filtration rate, carries a number of serious health consequences, including an increased risk of cardiovascular disease [6,18] anorexia, hyperparathyroidism, cancer, autoimmune diseases, and hyperhomocysteinemia (HHcy) [19].

Studies show that HHcy is a significant risk factor for the development of atherosclerotic processes that lead to cardiovascular disease (CVD) [6] and stroke [20]. Homocysteine (Hcy) levels higher than 20.0 μmol/L have been reported to increase the risk of death by 4.5 [19]. It is more likely that stage 3 patients will die from CVD than progress to ESRD. In addition, the mortality rate of patients with both CVD and CKD is higher than that of patients suffering from CVD alone [20].

The persistent elevation of parathyroid hormone (PTH) causes the development of osteitis fibrosa cystica and osteoarthritis [21]. In addition to the morbidity associated with bone disease, hyperparathyroidism is associated with systemic toxicity and increased serum calcium and phosphorus levels. Elevated concentrations of both of these elements are responsible for the development of vascular calcification, which is one of the mechanisms of increased morbidity and associated cardiovascular mortality observed in patients with CKD [22].

Kidneys are an important source of antioxidant enzymes, e.g., glutathione peroxidase, which is why pro-oxidant levels in CKD are elevated [23]. For this reason, dialysis or kidney transplantation can improve both the outcome and prognosis of ESRD [24].

Patients with CKD on hemodialysis (HD) are characterized by an excessive increase in oxidative stress, which is associated with progressive deterioration in kidney function and, consequently, higher mortality rates [25]. In addition, HD patients are at risk of malnutrition, cardiovascular complications, and all-cause mortality from oxidative stress and inflammation [1].

Some disorders, such as nutritional deficiencies, recurrent vomiting, appetite disorders and progressive anorexia, muscle cramps, bone diseases, and insomnia, can be minimized with appropriate diet and supplementation [4,26]. CKD clearly predisposes patients to disorders of vitamins and trace elements [1,7,8].

The guidelines of the European Society for Clinical Nutrition and Metabolism (ESPEN) recommend exact doses of vitamin supplements, which mostly correspond to the recommended daily dose for healthy adults. However, these guidelines on supplementation do not always coincide with the loss of vitamins during dialysis. Therefore, they will not always be appropriate for CKD patients. Due to the risk of toxicity with some vitamin levels being too high, care should be taken when supplementing [8,27] (Table 1). Nevertheless, there has been great variation in the prescription of supplements across countries, and practices regarding micronutrient supplementation are far from recommended [12].

## 3. The Effects of Vitamin D on Chronic Kidney Disease

**Figure 1:**
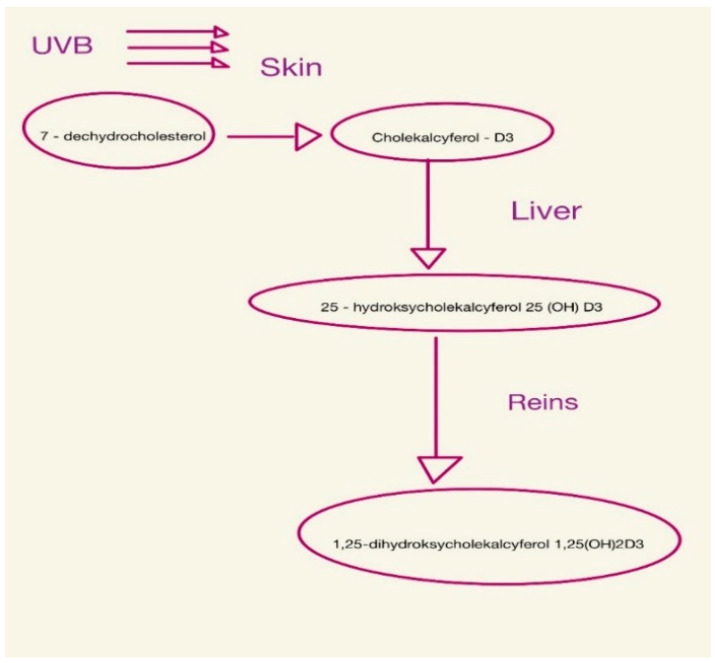
Consecutive steps of vitamin D synthesis.

Vitamin D is found in the body as 25-hydroxycholecalciferol or 1,25-dihydroxycholecalciferol (calcitriol) [28,29,30], as shown in Figure 1.

It is important in bone formation, immunity, vascular and nervous systems, and reproduction [30]. Vitamin D deficiency appears early in CKD and tends to worsen with progressive loss of kidney function [9,31,32]. This is because, from the early stages of CKD, there is an increase in serum levels of FGF-23 and PTH [33]. PTH increases calcium reabsorption in the kidneys and the excretion of phosphorus in the tubules, while also stimulating calcitriol synthesis [1,34]. FGF-23, on the other hand, inhibits the reabsorption of renal phosphorus and reduces the concentration of calcitriol in serum by inhibiting the renal enzyme 1-α-hydroxylase and stimulating the enzyme 24-hydroxylase, which is responsible for vitamin D catabolism [21,28]. For this reason, vitamin D deficiency (<20 ng/mL) and insufficiency (20–29 ng/mL) are common in patients with chronic kidney disease [9]. Filipov et al. showed that vitamin D deficiency in patients with CKD occurs in more than 80% of patients [35].

Vitamin D deficiency is associated with an increased risk of mortality and secondary hyperparathyroidism (SHPT) [30]. Since the glomerular filtration rate (GFR) decreases with impaired renal function, the ability to hydroxylate calcidiol to calcitriol decreases [8]. A deficiency in calcitriol, the active form of vitamin D, limits the intestinal absorption of calcium, which, combined with ongoing phosphate retention, contributes to the development of secondary hyperparathyroidism [26,36]. In addition, it is associated with reduced bone mineral density (BMD), muscle weakness, and an increased risk of falling, as well as an increased risk of cardiovascular disease, high blood pressure, diabetes, cancer, and autoimmune diseases [31,33].

In a meta-analysis of 10 prospective studies, Pilz et al. confirmed that the risk of death from any cause in patients with CKD increases by 14% for every 10 ng/mL reduction in vitamin D levels [37]. Therefore, the Kidney Disease Outcomes Quality Initiative (KDOQI) and Kidney Disease Improving Global Outcomes (KDIGO) group concluded that vitamin D deficiency should be avoided in patients with CKD on dialysis by using supplementation to prevent SHPT and other complications [9,29,38]. Many daily, weekly, or monthly vitamin D supplementation regimens with ergocalciferol (D2) or cholecalciferol (D3) have been recommended [21,36].

### Vitamin D Supplementation

The KDIGO group recommends vitamin D supplementation but does not specify which measures to take or the optimal strategy for restoring vitamin D levels [29,38]. Some studies suggest that serum 25(OH)D levels should be maintained at >75 nmol/L (30 ng/mL) in healthy individuals to prevent an increase in PTH levels [31]. Native vitamin D supplementation (ergocalciferol, cholecalciferol, and calcifediol) has been described in the literature as an alternative to calcitriol, but the effect of this supplementation on clinically relevant outcomes remains unexplained [21,31,36,39].

Many scientists have studied the effectiveness of cholecalciferol. Based on their results, it can be concluded that vitamin D supplementation in this form is effective and safe [8,30,34,40]. This was confirmed by Chandra et al., among others [41]. They examined the effectiveness of the weekly supplementation of cholecalciferol (vitamin D3) at increasing serum 25-hydroxyvitamin D levels and lowering PTH levels in patients with CKD. In this double-blind, randomized trial, people with CKD were randomly assigned to receive either 50,000 IU of cholecalciferol or a placebo once a week. At week 6, a significant difference was found between the treatment group and the placebo group (*p* = 0.001), and this difference persisted until the end of the study (12 weeks) [41]. Similar conclusions were reached by Tokmak et al. [42] and Okša et al. [33]. They showed that cholecalciferol supplementation is safe, well tolerated, and justified in replenishing vitamin D stores, with a higher dose being more effective and equally safe. Wissing et al. found that cholecalciferol supplementation does not prevent bone loss after kidney transplantation but contributes to the normalization of PTH levels [32]. However, in their study involving kidney transplant patients, Courbebaisse et al. showed that vitamin D deficiency persists 1 year after transplantation in the absence of treatment. They also showed that an increase in serum vitamin D 25-OH levels above 30 ng/mL requires a high dose of cholecalciferol, which improves secondary hyperparathyroidism after kidney transplantation. Importantly, it has been shown that this goal can be achieved without negative side effects [43]. Matias also showed that oral cholecalciferol supplementation reduces vitamin D deficiency, better controls mineral metabolism with less use of active vitamin D, weakens inflammation, reduces the dosage of erythropoiesis stimulants, and possibly improves cardiac dysfunction [44].

Ziyad Al-Aly’s study on the effects of ergocalciferol on serum concentrations of 25-hydroxyvitamin D and intact plasma parathyroid hormone shows that an increase in 25-hydroxyvitamin D levels greater than 5 ng/mL (12 nmol/L) is associated with a significant probability of a more than 30% decrease in intact plasma PTH levels. This means that the administration of ergocalciferol has a beneficial effect on PTH levels [9]. Blair et al. showed that ergocalciferol supplementation (50,000 IU/week × 24) was associated with a significant improvement from baseline in serum 25(OH)D concentrations. In addition, under the influence of supplementation, the level of glycosylated hemoglobin decreased while the hemoglobin level improved. The results suggest that supplementing your diet with ergocalciferol may help improve glycemic control in the treatment of diabetes [36]. They seem to agree that ergocalciferol supplementation appears to be a safe and effective treatment for the CKD population. It may raise serum 25(OH)D levels and reduce PTH levels [22,29,34,45], and it may have an epoetin-sparing effect [46].

Armas et al. compared the effectiveness of vitamin D2 with vitamin D3. They showed that vitamin D2 is one-third less effective than vitamin D3. Therefore, doctors should consider abandoning vitamin D2 supplementation in favor of the stronger vitamin D3 [31]. In contrast, Moe showed no significant difference in PTH reduction between patients treated with doxercalciferol and cholecalciferol [47].

There is growing evidence of the usefulness of vitamin D supplementation in dialysis patients, who are the most likely to be deficient in vitamin D. However, there is still no data indicating which dose would be most effective [21,26,36,40,48].

**Figure 2:**
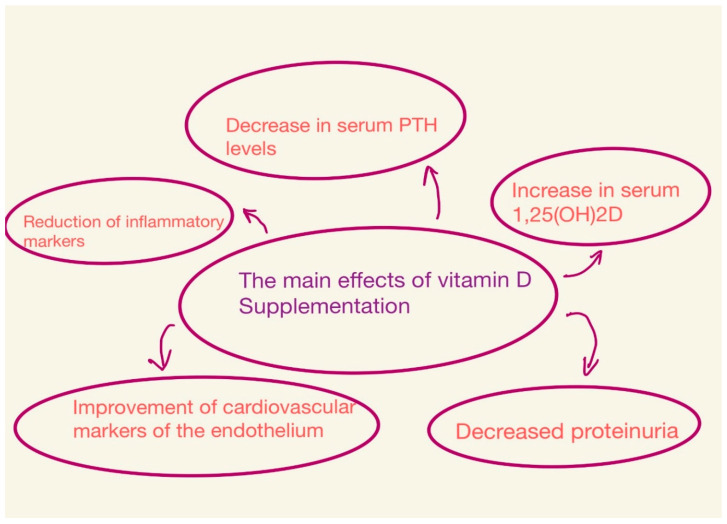
Main effects of vitamin D supplementation.

The effects depend on the dose of vitamin D, the type of vitamin D compounds, the duration of the study, and the population studied [21,34] (Figure 2).

Dogan et al. investigated the effect of monthly oral supplementation of 300,000 IU of vitamin D3 (cholecalciferol) on markers of uremic bone disease (UBD), such as PTH and alkalinophosphatase. In conclusion, the authors suggest that treatment with oral cholecalciferol results in a statistically significant reduction in serum PTH levels, but does not result in a statistically significant change in Ca or P [49]. Similar conclusions were reached by Pierre Delanaye et al. In his study on the effect of cholecalciferol (25,000 IU), administered every 2 weeks, he stated that cholecalciferol is effective and safe and does not adversely affect the levels of calcium, phosphorus, PTH, and vascular calcification [28].

Guillaume Jean sought to evaluate the efficacy and safety of a monthly dose of cholecalciferol (100,000 IU) in patients with a vitamin D (HD) deficiency undergoing hemodialysis. At the end of the study, of the 107 patients, 91% had serum 25(OH)D levels higher than the target level (>75 nmol/L). Serum calcitriol (1,25(OH)2D) levels also increased. However, no significant changes were observed regarding calcemia, phosphatemia, blood pressure, serum albumin, hemoglobin, and C-reactive protein. Based on this and other studies, it can be concluded that the long-term monthly administration of cholecalciferol orally is a safe and effective method for correcting vitamin D deficiency [30,48,50]. The most visible consequences of supplementation are a slight decrease in PTH and bone markers and an increase in 1,25(OH)2D in serum [8,26].

## 4. Effect and Supplementation of B Vitamins

B vitamins are very important for the proper functioning of the body. They are involved in metabolism and immune function and contribute to overall growth and development [7,13]. Currently, there are no clear indications regarding the supplementation of vitamins from this group, as studies of B vitamins in dialysis patients have yielded different results [38]. However, existing studies of poor intake and levels of B vitamins in adult patients have led to routine vitamin B supplementation in many countries [51].

The suspicion of the inadequate intake of B vitamins in patients with CKD, especially those on dialysis, is due to the fact that they have a lower appetite and a low protein supply in their diet [52]. At the same time, patients on dialysis because of uremia, due to some medications they take, have an increased need for certain B vitamins. Studies show that deficiencies in these vitamins occur directly as a result of dialysis [14].

It has been shown that the expression of thiamine and folic acid carriers is significantly reduced in an animal model of CKD [53]. Therefore, it can be concluded that the intestinal absorption of riboflavin, pyridoxine, and biotin in humans with CKD is also reduced [52].

### 4.1. Thiamine—B1

Thiamine is a hydrophilic B vitamin involved in many metabolic functions [54] that serves as a cofactor for oxidative decarboxylation reactions. The classic symptoms of thiamine deficiency are cardiomyopathy or Wernicke’s encephalopathy. Typically, these conditions are associated with alcohol abuse. However, in a few cases, this complication occurs in people on dialysis. Encephalopathy in these patients usually manifests itself only as impaired consciousness [55]. However, thiamine blood levels in patients with CKD are considered to be within the reference range of healthy subjects, or may even be increased. Thus, there are few studies on vitamin B1 supplementation [38]. Frank et al. evaluated patients with stage 4 and 5 CKD for thiamine intake. They showed that the average plasma thiamine concentration in this group of patients was 64.2 nmol/L, and a significant proportion of patients were deficient in vitamin B1. They pointed out that thiamine consumption in dialysis patients is lower than in healthy people. Due to the lack of some data, such as median values, it is difficult to compare the results of this study with others [54]. Some studies indicate that the bioactive vitamin B1 compound TDP is lost during dialysis. The amount of loss is related to the patient’s body weight but is not affected by vitamin B1 intake or the standard dose of supplementation [38].

In their cross-sectional study of 288 dialysis patients, Saka et al. assessed the relationship between thiamine levels in the blood and other clinical parameters. In total, 30 patients (12.4%) had lower blood thiamine levels than the lower normal limit. Thiamine blood levels correlated with age, body mass index, and Barthel score (BI). The proportion of patients with end-stage CKD with low thiamine blood levels is high. Low physical performance—a low BI score—is an independent risk factor for thiamine deficiency. The authors point out that physicians should be aware of the possible occurrence of thiamine deficiency in patients with end-stage CKD, especially those with low physical function [56].

Although the data do not indicate that all patients are thiamine deficient, there are indications that there is a risk of insufficient concentrations in patients with CKD [55]. It is also not known whether the daily intake level is sufficient in patients with CKD. Therefore, the daily supplementation of vitamin B1 seems reasonable to prevent possible deficiencies and their unpleasant consequences [38,56].

### 4.2. Riboflavin—B2

Riboflavin is a hydrophilic vitamin of group B. It is necessary for oxidation-reduction reactions [57]. After phosphorylation by adenosine triphosphate, riboflavin is converted to flavin mononucleotides (FMNs). Most FMNs are converted to flavin adenine dinucleotides (FADs) by FAD synthetase. Therefore, FADs are the dominant flavoenzymes in the body. The enzymes with which FMNs and FADs are associated with include oxygenases, monooxygenases, dehydrogenases, oxidoreductases, and electron transferases [58].

Porrini et al. studied patients with advanced CKD who were not on dialysis using the α-erythrocyte glutathione reductase (a-EGR) stimulation index to assess riboflavin counts. In this study, 8% of patients had elevated levels of a-EGR, indicating riboflavin deficiency. When the protein intake prescribed to these patients was intentionally reduced compared to their usual intake, the incidence of elevated a-EGR increased from 8% to 25% and 41% [59]. Therefore, several studies have recommended riboflavin supplements for patients with CKD, especially when they are on low-protein diets [13,38].

### 4.3. Niacin—B3

Niacin is a hydrophilic B vitamin that is consumed as a nicotinic acid amide from animal sources or in the form of nicotinic acid from plant sources. Niacin becomes active in humans when it is converted to nicotinamide adenine dinucleotides or nicotinamide adenine dinucleotide phosphate [60]. Niacin deficiency causes pellagra, a condition characterized by skin lesions, diarrhea, dementia, depression, and nausea; sometimes, the disease ends in death. Pellagra has been linked to chronic low-protein diets, alcoholism, anorexia, and intestinal malabsorption [61]. Therefore, it is believed that patients with CKD who are prescribed low-protein diets with phosphorus restriction may be at risk of niacin deficiency due to the low niacin content in plant foods [62].

Kang et al. found that because a low dose of niacin improves dyslipidemia, lowers serum phosphorus levels, and increases the GFR with rare adverse effects, low-dose supplementation may be considered in patients with CKD [63]. However, there are currently no high-quality clinical trials that have evaluated niacin supplementation in patients with CKD. Nicotinamide may be associated with side effects such as hot flashes, thrombocytopenia, hepatoxicity, gastrointestinal symptoms, and increased serum uric acid [61,63]. Recently, some studies have indicated that niacin supplementation with 400-1000 mg is necessary to appropriately lower phosphate levels [5]. Therefore, there is no hard evidence at this time to justify vitamin B3 supplementation. Patients with CKD with very low protein intake may consider supplementation at the recommended daily intake to prevent deficiencies while not exposing themselves to side effects [63].

### 4.4. Pyridoxine—B6

Vitamin B6 occurs in vivo as six compounds: pyridoxal, pyridoxine, and pyridoxamine, and as the 5′-phosphates of these three compounds. Pyridoxal-5-phosphate (PLP) is a cofactor for many enzymes, especially those involved in amino acid metabolism. PLP is necessary for (d)-aminolevulinate synthase to initiate heme synthesis [8,14].

Vitamin B6 losses during dialysis are still a matter of controversy [38]. A 35% decrease in pyridoxine levels was reported after a single dialysis session. On the other hand, since the vitamin is closely related to protein, its losses are considered somewhat moderate [52]. Vitamin deficiency was not observed in patients receiving 50 mg of pyridoxine after each dialysis session. Conversely, in those CKD patients who did not receive B6 supplementation, B6 deficiency was found in 78% of cases [14].

Kopple et al. and Podda et al. showed that many patients with CKD have suboptimal serum vitamin B6 levels [64,65]. Kopple et al. conducted both dietary and biochemical assessments of the pyridoxine status in patients at different stages of CKD. In a cross-sectional analysis, the amount of vitamin B6 consumed in food decreased with a decrease in the GFR. The average intake of vitamin B6 in patients with severe CKD was significantly lower than the dietary recommendations for their age group. This decreasing intake was reflected in the erythrocyte pyruvic aminotransferase (EGPT) stimulation rate. EGPT is an indicator of vitamin B6 deficiency. The study showed that the average EGPT stimulation rate increased inversely to the CKD stage. Patients with stage 3 and 4 CKD had higher GFRs than patients with stage 4 and 5 CKD. All of them were significantly higher than normal control values [64].

Many drugs and other compounds (including isoniazid, thyroxine, iproniazid, theophylline, hydralazine, caffeine, penicillamine, ethanol, and oral contraceptives) can interfere with the work or metabolism of pyridoxine and thus increase the likelihood of vitamin B6 deficiency. This is particularly likely in patients with CKD because they may often be prescribed these medications, and they show lower vitamin B6 intake with increased demand. These data suggest that patients with stage 3 or higher CKD are at an increased risk of vitamin B6 deficiency and should therefore take the appropriate supplementation to reduce CV risk [5,38]. Both the European Society for Parenteral and Enteral Nutrition and Australian Care guidelines for renal failure recommend that vitamin B6 be supplemented daily at a dose of 5 mg [59,64,65].

### 4.5. Cobalamin—B12

Cobalamin is a nutrient that plays a key role in human health. It is essential as a cofactor for enzyme methionine synthase and other biochemical reactions such as the beta-oxidation of fatty acids, DNA synthesis, and red blood cell production. Cobalamin is one of the most complex coenzymes in nature [19].

In healthy adults, vitamin B12 deficiency is extremely rare. Even if a healthy person consumed insufficient amounts of vitamin B12 for a period of 3 years, he would not have inadequate levels of this vitamin. However, these data do not necessarily apply to patients with CKD because there are no data on the amount of vitamin B12 stored in the body in patients with this disease [38]. Therefore, one can only rely on the suggestion that patients with CKD undergoing hemodialysis respond positively after supplementation with vitamin B12, even if plasma values are optimal [66]. This may be related to the fact that the amount of B12 in plasma is not a sensitive indicator of overall B12 levels [67]. For this vitamin, methylmalonic acid and homocysteine are more sensitive indicators. B12, like other B vitamins, is found in high-protein foods. Thus, patients who consume small amounts or stay on low-protein diets for long periods without supplementation may have insufficient vitamin B12 levels. Some authors believe that the available data do not indicate that all patients with CKD are deficient in this vitamin. However, it is prudent for patients on a low (0.6 g/day) or very low (0.3 g/day) protein diet to receive a vitamin B12 supplement [68].

In contrast, some authors state that vitamin B12 deficiency is a common feature of patients with CKD due to elevated concentrations of holotranscobalamin (TC2). The kidney plays an important role in the metabolism of TC2 because it is filtered into the glomeruli, and from there, it is reabsorbed into the proximal tubule. Defects in protein resorption in the proximal tubule can therefore lead to the biologically active loss of TC2 in the urine. Functional vitamin B12 deficiency has been observed despite normal total B12 levels, which may explain the increased leakage of TC2 in the urine, lower absorption of TC2 in the proximal tubule, and the lower cellular absorption of TC2 [69].

When deciding on supplementation, it is important to consider the fact that high levels of vitamin B12 can be harmful to people with CKD. This is related to the metabolism of cyanide, which is impaired in these individuals due to a reduced glomerular filtration rate. Cyanocobalamin, the most common form of vitamin B12 replacement, is metabolized to active methylcobalamin by releasing small amounts of cyanide. Under normal conditions, methylcobalamin binds to cyanide, converting it to harmless cyanocobalamin. However, in patients with CKD, this process is disturbed. In addition, excessive amounts of cyanocobalamin supplementation may release cyanide ions that are not excreted and contribute to complications such as uremic neuropathy [66,67].

### 4.6. Folic Acid—B9

Folic acid (FA) is pteroylmonoglutamic acid. FA deficiency causes megaloblastic anemia. Since the human body is unable to synthesize folic acid, it must be supplied through the diet [19]. Folic acid is derived from polyglutamates, which are converted to monoglutamates in the intestine and are then transported through the mucous epithelium [66].

The poor binding of FA to plasma proteins results in significant losses during each dialysis session. After a single procedure, a 37% decrease in plasma FA concentration was described. According to the available evidence, folate supplementation at a dose of 1 mg/day should prevent deficiencies in hemodialysis patients. Interestingly, increasing the dose to 2 mg/day results in a five-fold increase in plasma FA concentrations. High doses of folic acid have been used to reduce cardiovascular complications in patients with end-stage renal disease (ESRD) due to its effect on homocysteine methylation. However, the benefits of this practice have never been confirmed [20].

It has been shown that patients with CKD demonstrate anionic inhibition of the membrane transport of 5-MTHF with a simultaneous decrease in the intracellular rate of folate incorporation. Therefore, it is suggested that the level of folate measured in the blood of people with uremia does not reflect its intracellular use since its uptake is altered due to anionic inhibition [16]. In a randomized prospective study conducted on 341 dialysis patients, Cianciolo et al. found that 5-MTHF supplementation compared with FA treatment improved the survival rate of patients treated with 5-MTHF, although there was no difference in Hcy levels between the two treatment groups [19,20].

The benefits of folate supplementation in people with impaired renal function do not appear to correlate fully with a decrease in serum homocysteine (Hcy) levels [8]. The main cause of the onset of atherosclerosis, which increases the risk of cardiovascular diseases, is endothelial dysfunction [20]. Some studies indicate that high doses of FA (5 mg per day), alone or in combination with other B vitamins, appear to improve endothelial function. FA improves endothelial function by reducing endovascular oxidative stress. It also affects the intracellular production of superoxides by increasing the half-life of NO. Folate therapy reduces but does not normalize Hcy levels, which are often elevated in patients with CKD. The mechanisms of this resistance to folic acid have not yet been fully elucidated [16].

The minimum dose of folic acid to achieve Hcy reduction has been debated. ESRD patients without diabetes may respond to a daily dose of 5 mg FA, but diabetic patients with ESRD may need up to 15 mg to lower Hcy levels by more than 20% and to see benefits related to CVD risk, regardless of FA enhancement [20].

The DIVINE study examined the effects of high-dose therapy with FA (40 mg/day), vitamin B12 (1000 mg/day), and vitamin B6 (2 mg/day) in patients with diabetic nephropathy. It showed that this treatment regimen does not increase survival or slow the progression of ESRD. Instead, it leads to a higher incidence of cardiovascular events and a greater decrease in the eGFR. However, such results may be influenced by its suboptimal study design, such as the lack of division of patients with CKD and ESRD [52].

Another study looked at the effects of B12 and FA supplementation on CVD after 24 months of treatment. The results indicated that FA reduced the risk of CVD by 15%. Greater benefits were seen in those studies where treatment was longer than 24 months [16].

The role of folic acid and vitamin B12 supplementation in reducing mortality and preventing progression to ESRD has not yet been determined [8,19]. According to a meta-analysis by Heinz et al., Hcy emerged as a risk factor for cardiovascular disease and mortality in ESRD, particularly in those who do not receive supplemental FA. Prospective studies have shown that in patients with ESRD, an increase in Hcy concentration of 5 μmol/L is associated with a 7% increase in the risk of all-cause mortality and a 9% increase in the risk of cardiovascular disease. Hcy levels in these patients appear to have increased from 13 to 31 μmol/L due to supplementation with B vitamins in intervention studies. This was associated with a 27% reduction in the risk of cardiovascular disease, although mortality did not decrease [70]. At the same time, Heinz’s randomized, double-blind, multicenter study showed that the increased intake of folic acid, vitamin B12, and vitamin B6 did not reduce overall mortality and had no significant effect on the risk of cardiovascular events in patients with end-stage renal disease. The China Stroke Primary Prevention Trial (CSPPT) noted that age, baseline Hcy levels, FA enhancement of grains, B12 status, kidney function, comorbidities, and medications may modify the effects of folic acid and vitamin B12 on cardiovascular risk [71].

## 5. Vitamin C

Vitamin C, or ascorbic acid (AA), is capable of inhibiting the oxidation of other compounds by donating a maximum of two electrons. After donating one electron, ascorbic acid becomes a free radical known as semidehydroascorbic acid. After receiving the second electron, semidehydroascorbic acid is converted into dehydroascorbic acid [72]. Through this process, free radicals are scavenged in the body, after which they are oxidized, reducing the risk of cell damage. Because vitamin C has a protective effect on oxidative stress [73], Martins evaluated the effect of whey protein and vitamin C supplementation on biomarkers of oxidative stress in patients with chronic hemodialysis. The results suggest a pro-oxidative effect of vitamin C alone [74].

According to some studies, including Morena et al., AA loss during a single dialysis session was about 28%, with some results reaching as high as 60%. Based on these studies, it can be concluded that the loss of vitamin C during each dialysis session is significant [75]. Some authors explain the frequent occurrence of AA deficiency and reduced vitamin C intake as being due to a potassium-restricted diet and dialysis losses [73,76]. Bohm et al. measured the amount of vitamin C in hemodialysis and found that it ranged from 92 to 334 mg per treatment. This was associated with a 50% reduction in plasma ascorbic acid concentrations [77]. Zhang et al. showed that in 64% of 284 patients with HD and PD, plasma vitamin C levels were insufficient [78].

Over the years, indications for ascorbate supplementation have been formulated very carefully. This is due to oxalate, which is a metabolite of AA. A large amount in the body can cause hyperoxalemia, which can be considered a uremic toxin. Oxalate levels in HD patients are twice as high as in non-dialysis patients, and can be up to seven times higher when supplemented with vitamin C. Therefore, high doses of vitamin C are not recommended in patients with advanced CKD [73,76]. Canavese et al. found that administering 500 mg of vitamin C 1 time per week can increase serum oxalates levels to the threshold of supersaturation. They suggest that during vitamin C supplementation, plasma oxalate levels should be monitored, especially if supplementation is long-term [76]. Despite this, due to the reported deficiency of vitamin C in HD patients, some authors believe that vitamin C supplementation can improve health [79]. Thanks to its properties, it protects the structure of lipid membranes, proteins, and DNA from damage. In Sarandol’s study, vitamin D supplementation was shown to be a long-term parenteral supplementation. It was found that 500 mg of vitamin C improves lipoprotein oxidation in HD patients [80]. In a cross-sectional study, Zhang et al. found that vitamin C deficiency may play an important role in increased inflammation in dialysis patients [78].

One randomized, prospective trial looked at the effects of rutin and vitamin C compared to vitamin C alone on oxidative stress and inflammation in HD patients. It studied 105 HD patients that were divided into three groups. Group 1 received the rutin/vitamin C combination, group 2 received AA (1 g), and group 3 was the control group. At the beginning and end of the study, serum malondialdehyde (MDA), glutathione peroxidase (GPx), high-sensitivity C-reactive protein (hs-CRP), tumor necrosis factor α (TNF-α), lipid profile levels, and the erythrocyte sedimentation rate were evaluated. The authors showed that vitamin C significantly increased serum GPx in group 2 (*p* = 0.001) compared with a negligible score in the other groups. In addition, they reported that both vitamin C alone and the rutin/vitamin C combination showed a protective role against lipid peroxidation, as evidenced by reduced levels of MDA. Finally, rutin had a beneficial synergistic effect with vitamin C in reducing TG and TNF-α levels and increasing HDL-C levels [81].

In a prospective, randomized trial, Fumeron et al. evaluated the effects of oral vitamin C supplementation on oxidative and inflammatory markers in patients undergoing chronic hemodialysis. Based on the results, they concluded that vitamin C did not modify the assessed markers of oxidative stress or inflammation in HD patients [82].

## 6. Vitamin A

Vitamin A is a set of fat-soluble compounds classified as retinoids. People consume retinyl esters or carotenoids, which are considered precursors to vitamin A [8,83]. Retinyl esters can undergo conversions to form retinol, which can then be converted to retinal and then retinoic acid. Carotenoids include b-carotene, a-carotene, and b-cryptoxanthin, with b-carotene being the most common form. In plasma, retinol is largely bound to the aporetinol-binding protein (RBP 4) [84]. This equimolar complex binds to itself as a component of another molecule, prealbumin (also called transthyretin). It then binds to RBP cell surface receptors. Retinol is introduced into the cell, while the apo-RBP protein is released and catabolized by the kidneys. The physiological action of vitamin A is mediated by the nuclear retinoic acid receptor (RAR) and the retinoid X receptor (RXR), which belong to the same nuclear receptor superfamily as the vitamin D receptor [83,85].

Vitamin A is essential for normal night vision. It also plays a role in the immune response, the differentiation of epithelial cells, and the morphogenesis of organs, including the kidneys, and it has antioxidant properties. Large-scale clinical trials in the general population have not shown the benefit of consuming high doses of retinol and carotenoids in preventing cancer or cardiovascular disease [83]. Many authors have confirmed that dialysis patients have elevated plasma concentrations of total vitamin A, RBP, RBP-associated vitamin A, and free vitamin A. These changes are thought to be associated with altered RBP metabolism. With renal failure, the degradation of this protein is impaired [84].

Serum vitamin A concentrations are often increased in patients with advanced CKD. Potential mechanisms include reduced RBP catabolism. Frey et al. showed that RBP 4 isoforms (a major transporter of retinol in the blood) are increased in CKD, which may partly explain the elevated plasma concentrations in patients with CKD. National Health and Examination Survey III data showed a link between elevated serum creatinine and elevated vitamin A levels. This finding confirms previous studies that described elevated vitamin A levels in non-dialysis patients with CKD, ESRD patients, and kidney transplant recipients. Since serum vitamin A levels begin to increase with increasing creatinine, there appears to be no need to provide additional vitamin A to patients with CKD, except in some specific cases [85].

Espe et al. found a strong association between low levels of retinol and the risk of sudden cardiac death and fatal infection. One possible interpretation is that lower levels of retinol are a marker of inflammation [86].

In dialysis patients, plasma vitamin A levels are elevated, and vitamin A deficiency is rarely observed. In fact, even a small level of vitamin A supplementation can cause vitamin A toxicity. Therefore, the guidelines for vitamin A supplementation recommend not exceeding the daily dose in healthy people (i.e., 700–900 lg DRE) [83,86].

## 7. Vitamin E

Vitamin E is a lipophilic molecule that is typically found in cell membranes. It comes in four forms: a-tocopherol, b-tocopherol, g-tocopherol, and d-tocopherol [8,87].

The role of oxidative stress as a pathological factor in several disease states is becoming increasingly apparent, and vitamin E supplementation is also being considered as a potential treatment for this condition [88,89,90] due to its antioxidant properties. Plasma vitamin E levels in patients with CKD do not appear to differ from healthy controls, even when vitamin E intake is reduced [83]. The metabolite of vitamin E, carboxyethyl hydroxychroman (CEHC), increases significantly in serum with deteriorating renal function [87]. Galli et al. suggest that CEHC accumulation may interfere with vitamin E in patients with uremic CKD. The results of clinical trials evaluating the effectiveness of vitamin E in the prevention of cardiovascular disease in people with CKD were mixed [89]. Mann et al. examined the outcomes in patients with mild to moderate renal failure and an increased risk of cardiovascular events who were given 400 IU/day of vitamin E as part of the Heart Outcomes Prevention Evaluation (HOPE) study [91]. According to studies involving patients who did not have CKD, there was no cardiovascular benefit from vitamin E supplementation. Additionally, the long-term use of vitamin E at a dose of 400 IU per day may cause an increased incidence of heart failure and hospitalizations associated with heart failure [88]. Only a randomized, prospectively controlled SPACE trial in 196 HD patients showed that oral vitamin E at 800 IU per day significantly reduced the risk of myocardial infarction, ischemic stroke, peripheral vascular disease, and unstable angina in patients with pre-existing cardiovascular disease. The results of the SPACE study have never been confirmed in any other study [89,91]. A study by Lonn et al., called HOPE-TOO, found that vitamin E supplements were associated with an increased incidence of heart failure. Thus, these studies suggest that among people at a high risk for cardiovascular events, vitamin E supplementation may not be advisable [88,92].

According to some studies, in non-diabetic patients with mild to moderate CKD, a treatment strategy with pravastatin, vitamin E, and Hcy reduction therapy (vitamin B12 and folic acid) leads to a significant reduction in the progression of carotid stenosis and a significant improvement in endothelial function and urinary albumin excretion. However, no significant effect on the eGFR was observed [90].

In HD patients and peritoneal dialysis (PD), serum a-tocopherol levels can range from low or normal to increased [87].

Numerous studies have shown that vitamin E supplements reduce oxidative stress [4,83,91].

## 8. Vitamin K

Vitamin K participates in the post-translational carboxylation of specific glutamic acid (Gla) residues in proteins (such as blood coagulation proteins and osteocalcin), allowing it to bind to calcium and interact with other compounds. This is necessary for blood coagulation and bone mineralization [93]. Vitamin K is available in two forms, vitamin K1 (phylloquinone) and vitamin K2 (menaquinone) [8,11]. The dietary form of vitamin K is phylloquinone, which is absorbed in the jejunum and ileum, and is stored mainly in the liver [11]. Bacteria in the gut produce vitamin K in the form of menaquinones, which are absorbed from the distal intestine and are also stored in the liver. If vitamin K deficiency occurs, proteins in the body may be insufficiently carboxylated [93,94].

Since vitamin K binds to lipoproteins, no loss is expected during the dialysis procedure [95]. Initial reports of serum vitamin K levels in dialysis patients were contradictory. However, recent discoveries of an increased non-carboxylated fraction of vitamin K-dependent proteins such as prothrombin (also referred to as vitamin K-induced protein or antagonism factor II, PIVKA-II) and osteocalcin have confirmed functional vitamin K deficiency in many dialysis patients [11,96]. Antibiotics that inhibit the gut flora and thus the bacterial production of vitamin K may increase the risk of vitamin K deficiency and impaired blood clotting. This is especially likely if the patient does not eat or take vitamin supplements, and therefore has a low intake of vitamin K [11]. In one study of hospitalized patients with a prolonged prothrombin time, one-third had CKD and were not on dialysis. A recent study of 172 patients with stage 3–5 CKD found that, depending on the vitamin K index used, between 6% and 97% of patients were deficient in this vitamin. When phylloquinone serum was used as the measure, there was a 6% deficiency in this population. When measuring vitamin K-II induced proteins (PIVKA-II), a less commonly used but potentially very accurate marker, 97% of patients were deficient [94,97]. In a study conducted by Elliott on 44 hemodialysis patients, the levels of vitamin K were measured in fasting blood samples. The study found that 13.6% of the subjects met the criteria for subclinical vitamin K deficiency (phylloquinone < 0.4 nmol/L), 51% for uncarboxylated osteocalcin (%ucOC > 20%), and 90.9% for PIVKA-II (>2.0 nmol/L). These results suggest that men and women with CKD should consume at least 90 mg per day of vitamin K [94].

Until recently, routine vitamin K supplementation was not recommended in dialysis patients. However, it is now possible to identify functional vitamin K deficiency by measuring non-carboxylated fractions of vitamin K-dependent proteins [11]. More recently, Westenfeld et al. were able to reduce dephosphorylated, non-carboxylated MGP with vitamin K2 supplements. This raises the possibility of a new therapy to prevent vascular calcification [98].

Some studies suggest that a high menaquinone intake in the general population has a beneficial effect on CVD and bone density, leading to a lower risk of bone fracture. However, the results are not conclusive [95]. A study of 4800 patients in Rotterdam found that a reduced amount of vitamin K2 in the diet was associated with a higher risk of aortic calcification, CV incidence, and mortality in the general population. Slowing the progression of calcification is a potential beneficial effect of vitamin K2 supplementation. On the other hand, Fulton et al. did not show a significant effect of vitamin K2 supplementation on vascular health in older adults with vascular disease. However, this study had several limitations, one of which was that patients received only 100 μg of vitamin K2. There are also reports that vitamin K2 supplementation may affect metabolic processes since it increases the insulin sensitivity index and lowers fasting glucose and 2 h glucose after OGTT [96]. On the other hand, there are also reports that show no effect on insulin resistance. Many well-designed, randomized trials are needed to determine with certainty appropriate recommendations for vitamin K supplementation [11,98].

## 9. Conclusions

Knowledge about the need for vitamin supplementation among patients with CKD remains incomplete [7,27]. The studies discussed in this paper suggest that it is very likely that patients with CKD are exposed to a vitamin deficiency, and supplementation should be considered as part of the treatment process [6,12]. Most authors agree that there is sufficient evidence that vitamin deficiencies in CKD include vitamins B1, B2, B6, C, K, and D [7,9,38,57,59,94]. However, some studies are of low quality. Hence, the recommendations of the KDOQI from 2020 point to the fact that there is a lack of high-quality studies with unanimous results. There is a risk of vitamin excess and toxicity which has to be taken into account when supplementation is planned. According to the authors of the KDOQI, there is no consensus and no clear answer regarding dosage, duration of supplementation or the type of vitamin D used, and the optimal dosage of vitamins C and E, and there is no clear explanation as to why vitamin K deficiency is more common in patients on dialysis [38].

The topic of vitamin A, E, and K supplementation remains controversial. Some papers suggest that supplementation should be undertaken and is beneficial. On the other hand, because of possible toxicity, some suggest the opposite. This leads to cautious recommendations for supplementation with these vitamins [8,11,38,83].

Based on current evidence, the individualized approach towards vitamin supplementation is recommended. Folic acid should be supplemented cautiously because a high intake may mask signs of anemia and progressive neurological disorders. Further research is needed to precisely define the demand for these essential nutrients in patients with CKD [9,27,95].

## Figures and Tables

**Table 1:** The information on selected vitamins’ daily supplementation in patients with CKD on HD [8].

Vitamins	HD Losses	European Guidelines for HD Patients	Risk of Toxicity
D	No	No	Yes
B1	13–40 mL/min	1.1–1.2 mg	No
B2	27–52 mL/min	1.1–1.3 mg	No
B3	Very low	14–16 mg	No data
B6	54–173 mL/min	10 mg	Yes
B9	135 mL/min	1 mg	Yes
B12	Controversial	2.4 lg	No data
C	80–280 mg/session	75–90 mg	Yes
A	No	No	Yes
E	No	400–800 IU	Possible
K	No data	No	No

## Data Availability

Data were derived from public domain resources.

## References

[1] Kestenbaum B., Belozeroff V. (2007). Mineral Metabolism Disturbances in Patients with Chronic Kidney Disease. Eur. J. Clin. Investig..

[2] Kovesdy C.P., Anderson J.E., Kalantar-Zadeh K. (2007). Paradoxical Association between Body Mass Index and Mortality in Men with CKD Not Yet on Dialysis. Am. J. Kidney Dis..

[3] Kalantar-Zadeh K., Kopple J.D., Kilpatrick R.D., McAllister C.J., Shinaberger C.S., Gjertson D.W., Greenland S. (2005). Association of Morbid Obesity and Weight Change over Time with Cardiovascular Survival in Hemodialysis Population. Am. J. Kidney Dis..

[4] Karamouzis I., Sarafidis P.A., Karamouzis M., Iliadis S., Haidich A.B., Sioulis A., Triantos A., Vavatsi-Christaki N., Grekas D.M. (2008). Increase in Oxidative Stress but Not in Antioxidant Capacity with Advancing Stages of Chronic Kidney Disease. Am. J. Nephrol..

[5] Kędzierska-Kapuza K., Szczuko U., Stolińska H., Bakaloudi D.R., Wierzba W., Szczuko M. (2023). Demand for Water-Soluble Vitamins in a Group of Patients with CKD versus Interventions and Supplementation—A Systematic Review. Nutrients.

[6] Schlieper G., Schurgers L., Brandenburg V., Reutelingsperger C., Floege J. (2016). Vascular Calcification in Chronic Kidney Disease: An Update. Nephrol. Dial. Transplant..

[7] Coveney N., Polkinghorne K.R., Linehan L., Corradini A.M., Kerr P.G. (2011). Water-Soluble Vitamin Levels in Extended Hours Hemodialysis. Hemodial. Int..

[8] Chazot C., Jean G., Kopple J.D. (2016). Can Outcomes Be Improved in Dialysis Patients by Optimizing Trace Mineral, Micronutrient, and Antioxidant Status?: The Impact of Vitamins and Their Supplementation. Semin. Dial..

[9] Al-Aly Z., Qazi R.A., González E.A., Zeringue A., Martin K.J. (2007). Changes in Serum 25-Hydroxyvitamin D and Plasma Intact PTH Levels Following Treatment with Ergocalciferol in Patients with CKD. Am. J. Kidney Dis..

[10] Steiber A.L., Kopple J.D. (2011). Vitamin Status and Needs for People with Stages 3-5 Chronic Kidney Disease. J. Ren. Nutr..

[11] de Vriese A.S., Caluwé R., Pyfferoen L., de Bacquer D., de Boeck K., Delanote J., de Surgeloose D., van Hoenacker P., van Vlem B., Verbeke F. (2020). Multicenter Randomized Controlled Trial of Vitamin K Antagonist Replacement by Rivaroxaban with or without Vitamin K2 in Hemodialysis Patients with Atrial Fibrillation: The Valkyrie Study. J. Am. Soc. Nephrol..

[12] Fissell R.B., Bragg-Gresham J.L., Gillespie B.W., Goodkin D.A., Bommer J., Saito A., Akiba T., Port F.K., Young E.W. (2004). International Variation in Vitamin Prescription and Association with Mortality in the Dialysis Outcomes and Practice Patterns Study (DOPPS). Am. J. Kidney Dis..

[13] Descombes E., Hanck A.B., Fellay G. (1993). Water Soluble Vitamins in Chronic Hemodialysis Patients and Need for Supplementation. Kidney Int..

[14] Corken M., Porter J. (2011). Is Vitamin B 6 Deficiency an Under-Recognized Risk in Patients Receiving Haemodialysis? A Systematic Review: 2000–2010. Nephrology.

[15] La Russa D., Pellegrino D., Montesanto A., Gigliotti P., Perri A., Russa A.L., Bonofiglio R. (2019). Oxidative Balance and Inflammation in Hemodialysis Patients: Biomarkers of Cardiovascular Risk?. Oxidative Med. Cell. Longev..

[16] Cianciolo G., De Pascalis A., Di Lullo L., Ronco C., Zannini C., La Manna G. (2017). Folic Acid and Homocysteine in Chronic Kidney Disease and Cardiovascular Disease Progression: Which Comes First?. Cardiorenal Med..

[17] Hill N.R., Fatoba S.T., Oke J.L., Hirst J.A., O’Callaghan C.A., Lasserson D.S., Hobbs F.D.R. (2016). Global Prevalence of Chronic Kidney Disease—A Systematic Review and Meta-Analysis. PLoS ONE.

[18] Fried L.F., Shlipak M.G., Crump C., Bleyer A.J., Gottdiener J.S., Kronmal R.A., Kuller L.H., Newman A.B. (2003). Renal Insufficiency as a Predictor of Cardiovascular Outcomes and Mortality in Elderly Individuals. J. Am. Coll. Cardiol..

[19] Angelini A., Cappuccilli M.L., Magnoni G., Chiocchini A.L.C., Aiello V., Napoletano A., Iacovella F., Troiano A., Mancini R., Capelli I. (2021). The link between homocysteine, folic acid and vitamin B12 in chronic kidney disease. G. Ital. Nefrol..

[20] Cianciolo G., La Manna G., Colì L., Donati G., D’Addio F., Persici E., Comai G., Wratten M., Dormi A., Mantovani V. (2008). 5-Methyltetrahydrofolate Administration Is Associated with Prolonged Survival and Reduced Inflammation in ESRD Patients. Am. J. Nephrol..

[21] Bhan I., Thadhani R. (2009). Vitamin D Therapy for Chronic Kidney Disease. Semin. Nephrol..

[22] Zisman A.L., Hristova M., Ho L.T., Sprague S.M. (2007). Impact of Ergocalciferol Treatment of Vitamin D Deficiency on Serum Parathyroid Hormone Concentrations in Chronic Kidney Disease. Am. J. Nephrol..

[23] Kelly D.M., Rothwell P.M. (2020). Prevention and Treatment of Stroke in Patients with Chronic Kidney Disease: An Overview of Evidence and Current Guidelines. Kidney Int..

[24] Eckardt K.U., Bansal N., Coresh J., Evans M., Grams M.E., Herzog C.A., James M.T., Heerspink H.J.L., Pollock C.A., Stevens P.E. (2018). Improving the Prognosis of Patients with Severely Decreased Glomerular Filtration Rate (CKD G4+): Conclusions from a Kidney Disease: Improving Global Outcomes (KDIGO) Controversies Conference. Kidney Int..

[25] Liakopoulos V., Roumeliotis S., Gorny X., Dounousi E., Mertens P.R. (2017). Oxidative Stress in Hemodialysis Patients: A Review of the Literature. Oxidative Med. Cell. Longev..

[26] Kandula P., Dobre M., Schold J.D., Schreiber M.J., Mehrotra R., Navaneethan S.D. (2011). Vitamin D Supplementation in Chronic Kidney Disease: A Systematic Review and Meta-Analysis of Observational Studies and Randomized Controlled Trials. Clin. J. Am. Soc. Nephrol..

[27] Jankowska M., Rutkowski B., Dębska-Ślizień A. (2017). Vitamins and Microelement Bioavailability in Different Stages of Chronic Kidney Disease. Nutrients.

[28] Delanaye P., Weekers L., Warling X., Moonen M., Smelten N., Médart L., Krzesinski J.M., Cavalier E. (2013). Cholecalciferol in Haemodialysis Patients: A Randomized, Double-Blind, Proof-of-Concept and Safety Study. Nephrol. Dial. Transplant..

[29] DeVille J., Thorp M.L., Tobin L., Gray E., Johnson E.S., Smith D.H. (2006). Effect of Ergocalciferol Supplementation on Serum Parathyroid Hormone and Serum 25-Hydroxyvitamin D in Chronic Kidney Disease. Nephrology.

[30] Jean G., Terrat J.C., Vanel T., Hurot J.M., Lorriaux C., Mayor B., Chazot C. (2008). Evidence for Persistent Vitamin D 1-Alpha-Hydroxylation in Hemodialysis Patients: Evolution of Serum 1,25-Dihydroxycholecalciferol after 6 Months of 25-Hydroxycholecalciferol Treatment. Nephron Clin. Pr..

[31] Armas L.A.G., Hollis B.W., Heaney R.P. (2004). Vitamin D2 Is Much Less Effective than Vitamin D3 in Humans. J. Clin. Endocrinol. Metab..

[32] Wissing K.M., Broeders N., Moreno-Reyes R., Gervy C., Stallenberg B., Abramowicz D. (2005). A Controlled Study of Vitamin D3 to Prevent Bone Loss in Renal-Transplant Patients Receiving Low Doses of Steroids. Transplantation.

[33] Okša A., Spustová V., Krivošíková Z., Gazdíková K., Fedelešová V., Lajdová I., Štefíková K., Bernasovská G., Žilinská Z., Dzúrik R. (2008). Effects of Long-Term Cholecalciferol Supplementation on Mineral Metabolism and Calciotropic Hormones in Chronic Kidney Disease. Kidney Blood Press. Res..

[34] Ravani P., Malberti F., Tripepi G., Pecchini P., Cutrupi S., Pizzini P., Mallamaci F., Zoccali C. (2009). Vitamin D Levels and Patient Outcome in Chronic Kidney Disease. Kidney Int..

[35] Filipov J.J., Zlatkov B.K., Dimitrov E.P., Svinarov D. (2015). Relationship between Vitamin D Status and Immunosuppressive Therapy in Kidney Transplant Recipients. Biotechnol. Biotechnol. Equip..

[36] Blair D., Byham-Gray L., Lewis E., McCaffrey S. (2008). Prevalence of Vitamin D [25(OH)D] Deficiency and Effects of Supplementation with Ergocalciferol (Vitamin D2) in Stage 5 Chronic Kidney Disease Patients. J. Ren. Nutr..

[37] Pilz S., Iodice S., Zittermann A., Grant W.B., Gandini S. (2011). Vitamin D Status and Mortality Risk in CKD: A Meta-Analysis of Prospective Studies. Am. J. Kidney Dis..

[38] Alp Ikizler T., Burrowes J.D., Byham-Gray L.D., Campbell K.L., Carrero J.-J., Chan W., Fouque D., Friedman A.N., Ghaddar S., Jordi Goldstein-Fuchs D. (2020). Kdoqi clinical practice guideline for nutrition in ckd: 2020 update. Am. J. Kidney Dis..

[39] Thadhani R. (2009). Is Calcitriol Life-Protective for Patients with Chronic Kidney Disease?. J. Am. Soc. Nephrol..

[40] Holick M.F., Biancuzzo R.M., Chen T.C., Klein E.K., Young A., Bibuld D., Reitz R., Salameh W., Ameri A., Tannenbaum A.D. (2008). Vitamin D2 Is as Effective as Vitamin D3 in Maintaining Circulating Concentrations of 25-Hydroxyvitamin D. J. Clin. Endocrinol. Metab..

[41] Chandra P., Nilo J., Binongo G., Ziegler T.R., Schlanger L.E., Wang W., Someren J.T., Tangpricha V. (2008). Cholecalciferol (vita min d 3) therapy and vitamin d insufficiency in patients with chronic kidney disease: A randomized controlled pilot study. Endocr. Pract..

[42] Tokmak F., Quack I., Schieren G., Sellin L., Rattensperger D., Holland-Letz T., Weiner S.M., Rump L.C. (2008). High-Dose Cholecalciferol to Correct Vitamin D Deficiency in Haemodialysis Patients. Nephrol. Dial. Transplant..

[43] Courbebaisse M., Thervet E., Souberbielle J.C., Zuber J., Eladari D., Martinez F., Mamzer-Bruneel M.F., Urena P., Legendre C., Friedlander G. (2009). Effects of Vitamin D Supplementation on the Calcium-Phosphate Balance in Renal Transplant Patients. Kidney Int..

[44] Matias P.J., Jorge C., Ferreira C., Borges M., Aires I., Amaral T., Gil C., Cortez J., Ferreira A. (2010). Cholecalciferol Supplementation in Hemodialysis Patients: Effects on Mineral Metabolism, Inflammation, and Cardiac Dimension Parameters. Clin. J. Am. Soc. Nephrol..

[45] Shah N., Bernardini J., Piraino B. (2005). Prevalence and correction of 25(oh) vitamin d deficiency in peritoneal dialysis patients. Perit. Dial. Int..

[46] Saab G., Young D.O., Gincherman Y., Giles K., Norwood K., Coyne D.W. (2007). Prevalence of Vitamin D Deficiency and the Safety and Effectiveness of Monthly Ergocalciferol in Hemodialysis Patients. Nephron Clin. Pr..

[47] Moe S.M., Saifullah A., LaClair R.E., Usman S.A., Yu Z. (2010). A Randomized Trial of Cholecalciferol versus Doxercalciferol for Lowering Parathyroid Hormone in Chronic Kidney Disease. Clin. J. Am. Soc. Nephrol..

[48] Jean G., Souberbielle J.C., Chazot C. (2009). Monthly Cholecalciferol Administration in Haemodialysis Patients: A Simple and Efficient Strategy for Vitamin D Supplementation. Nephrol. Dial. Transplant..

[49] Dogan E., Erkoc R., Sayarlioglu H., Soyoral Y., Dulger H. (2008). Effect of Depot Oral Cholecalciferol Treatment on Secondary Hyperparathyroidism in Stage 3 and Stage 4 Chronic Kidney Diseases Patients. Ren. Fail..

[50] Jean G., Souberbielle J.C., Chazot C. (2017). Vitamin D in Chronic Kidney Disease and Dialysis Patients. Nutrients.

[51] Don T., Friedlander S., Wong W. (2010). Dietary Intakes and Biochemical Status of B Vitamins in a Group of Children Receiving Dialysis. J. Ren. Nutr..

[52] House A.A., Eliasziw M., Cattran D.C., Churchill D.N., Oliver M.J., Fine A., Dresser G.K., David Spence J. (2010). Effect of B-Vitamin Therapy on Progression of Diabetic Nephropathy A Randomized Controlled Trial. JAMA.

[53] Bukhari F.J., Moradi H., Gollapudi P., Ju Kim H., Vaziri N.D., Said H.M. (2011). Effect of Chronic Kidney Disease on the Expression of Thiamin and Folic Acid Transporters. Nephrol. Dial. Transplant..

[54] Frank T., Czeche K., Bitsch R., Stein G. (2000). Assessment of Thiamin Status in Chronic Renal Failure, Transplant Recipients and Hemodialysis Patients a Multivitamin. Int. J. Vitam. Nutr. Res..

[55] Hung S.C., Hung S.H., Tarng D.C., Yang W.C., Chen T.W., Huang T.P. (2001). Thiamine Deficiency and Unexplained Encephalopathy in Hemodialysis and Peritoneal Dialysis Patients. Am. J. Kidney Dis..

[56] Saka Y., Naruse T., Kato A., Tawada N., Noda Y., Mimura T., Watanabe Y. (2018). Thiamine Status in End-Stage Chronic Kidney Disease Patients: A Single-Center Study. Int. Urol. Nephrol..

[57] Suwannasom N., Kao I., Pruß A., Georgieva R., Bäumler H. (2020). Riboflavin: The Health Benefits of a Forgotten Natural Vitamin. Int. J. Mol. Sci..

[58] Fischer M., Bacher A. (2008). Biosynthesis of Vitamin B2: Structure and Mechanism of Riboflavin Synthase. Arch. Biochem. Biophys..

[59] Porrini M., Simonetti P., Ciappellano S., Testolin G., Gentile M., Manna G., Fellin G., D’Amico G. (1989). Thiamin, Riboflavin and Pyridoxine Status in Chronic Renal Insufficiency. Int. J. Vitam. Nutr. Res..

[60] Berns J.S. (2008). Niacin and Related Compounds for Treating Hyperphosphatemia in Dialysis Patients. Semin. Dial..

[61] Rennick A., Kalakeche R., Seel L., Shepler B. (2013). Nicotinic Acid and Nicotinamide: A Review of Their Use for Hyperphosphatemia in Dialysis Patients. Pharmacotherapy.

[62] Cheng S.C., Young D.O., Huang Y., Delmez J.A., Coyne D.W. (2008). A Randomized, Double-Blind, Placebo-Controlled Trial of Niacinamide for Reduction of Phosphorus in Hemodialysis Patients. Clin. J. Am. Soc. Nephrol..

[63] Kang H.J., Kim D.K., Lee S.M., Kim K.H., Han S.H., Kim K.H., Kim S.E., Son Y.K., An W.S. (2013). Effects of Low-Dose Niacin on Dyslipidemia and Serum Phosphorus in Patients with Chronic Kidney Disease. Kidney Res. Clin. Pract..

[64] Kopple J.D., Mercurio K., Blumenkrantz M.J., Jones M.R., Tallos J., Roberts C., Card B., Saltzman R., Casciato D.A., Swendseid M.E. (1981). Daily Requirement for Pyridoxine Supplements in Chronic Renal Failure. Kidney Int..

[65] Podda G.M., Lussana F., Moroni G., Faioni E.M., Lombardi R., Fontana G., Ponticelli C., Maioli C., Cattaneo M. (2007). Abnormalities of Homocysteine and B Vitamins in the Nephrotic Syndrome. Thromb. Res..

[66] Capelli I., Cianciolo G., Gasperoni L., Zappulo F., Tondolo F., Cappuccilli M., La Manna G. (2019). Folic Acid and Vitamin B12 Administration in CKD, Why Not?. Nutrients.

[67] Wu H.H.L., Wang A.Y.M. (2022). Vitamin B12 and Chronic Kidney Disease. Vitam. Horm..

[68] Jamison R.L., Hartigan P., Kaufman J.S., Goldfarb D.S., Warren S.R., Guarino P.D., Gaziano J.M. (2007). Effect of Homocysteine Lowering on Mortality and Vascular Disease in Advanced Chronic Kidney Disease and End-Stage Renal Disease A Randomized Controlled Trial. JAMA.

[69] Dastidar R., Sikder K. (2022). Diagnostic Reliability of Serum Active B12 (Holo-Transcobalamin) in True Evaluation of Vitamin B12 Deficiency: Relevance in Current Perspective. BMC Res. Notes.

[70] Heinz J., Kropf S., Domröse U., Westphal S., Borucki K., Luley C., Neumann K.H., Dierkes J. (2010). B Vitamins and the Risk of Total Mortality and Cardiovascular Disease in End-Stage Renal Disease: Results of a Randomized Controlled Trial. Circulation.

[71] Huo Y., Li J., Qin X., Huang Y., Wang X., Gottesman R.F., Tang G., Wang B., Chen D., He M. (2015). Efficacy of Folic Acid Therapy in Primary Prevention of Stroke among Adults with Hypertension in China: The CSPPT Randomized Clinical Trial. JAMA J. Am. Med. Assoc..

[72] Deicher R., Hörl W.H. (2003). Vitamin C in Chronic Kidney Disease and Hemodialysis Patients. Kidney Blood Press. Res..

[73] Chaghouri P., Maalouf N., Peters S.L., Nowak P.J., Peczek K., Zasowska-Nowak A., Nowicki M. (2021). Two Faces of Vitamin C in Hemodialysis Patients: Relation to Oxidative Stress and Inflammation. Nutrients.

[74] Martins M.L., da Silva A.T., Machado R.P., Ramos H.P., Martinelli C., Silveira T.T., da Silva E.L., Wazlawik E. (2021). Vitamin C Decreases Reduced Glutathione in Chronic Haemodialysis Patients: A Pilot, Randomised, Double-Blind Trial. Int. Urol. Nephrol..

[75] Morena M., Cristol J.-P., Bosc J.-Y., Tetta C., Forret G., Leger C.-L., Delcourt C., Papoz L., Descomps B., Canaud B. (2002). Convective and Diffusive Losses of Vitamin C during Haemodiafiltration Session: A Contributive Factor to Oxidative Stress in Haemodialysis Patients. Nephrol. Dial. Transplant..

[76] Canavese C., Petrarulo M., Massarenti P., Berutti S., Fenoglio R., Pauletto D., Lanfranco G., Bergamo D., Sandri L., Marangella M. (2005). Long-Term, Low-Dose, Intravenous Vitamin C Leads to Plasma Calcium Oxalate Supersaturation in Hemodialysis Patients. Am. J. Kidney Dis..

[77] Böhm F., Tiroke K., Schneider S., Sperschneider H., Stein G., Bitsch R. (1997). Vitamin C Status of Patients with Chronic Renal Failure, Dialysis Patients and Patients after Renal Transplantation. Int. J. Vitam. Nutr. Res..

[78] Zhang K., Liu L., Cheng X., Dong J., Geng Q., Zuo L. (2011). Low Levels of Vitamin C in Dialysis Patients Is Associated with Decreased Prealbumin and Increased C-Reactive Protein. BMC Nephrol..

[79] Raimann J.G., Levin N.W., Craig R.G., Sirover W., Kotanko P., Handelman G. (2013). Is Vitamin C Intake Too Low in Dialysis Patients?. Semin. Dial..

[80] Sarandol E., Erdinc S., Senol E., Ersoy A., Surmen-Gur E. (2022). Effects of Vitamin C Supplementation on Oxidative Stress and Serum Paraoxonase/Arylesterase Activities in Patients on Long-Term Hemodialysis. Nefrologia.

[81] Omar S., El Borolossy R.M., Elsaid T., Sabri N.A. (2022). Evaluation of the Combination Effect of Rutin and Vitamin C Supplementation on the Oxidative Stress and Inflammation in Hemodialysis Patients. Front. Pharm..

[82] Fumeron C., Nguyen-Khoa T., Saltiel C., Kebede M., Buisson C., Drüeke T.B., Lacour B., Massy Z.A. (2005). Effects of Oral Vitamin C Supplementation on Oxidative Stress and Inflammation Status in Haemodialysis Patients. Nephrol. Dial. Transplant..

[83] Rojo-Trejo M.H., Robles-Osorio M.L., Sabath E. (2022). Liposoluble Vitamins A and E in Kidney Disease. World J. Nephrol..

[84] Manickavasagar B., McArdle A.J., Yadav P., Shaw V., Dixon M., Blomhoff R., O’Connor G., Rees L., Ledermann S., van’t Hoff W. (2014). Hypervitaminosis A Is Prevalent in Children with CKD and Contributes to Hypercalcemia. Pediatr. Nephrol..

[85] Frey S.K., Nagl B., Henze A., Raila J., Schlosser B., Berg T., Tepel M., Zidek W., Weickert M.O., Pfeiffer A.F.H. (2008). Isoforms of Retinol Binding Protein 4 (RBP4) Are Increased in Chronic Diseases of the Kidney but Not of the Liver. Lipids Health Dis..

[86] Espe K.M., Raila J., Henze A., Krane V., Schweigert F.J., Hocher B., Wanner C., Drechsler C. (2011). Impact of Vitamin A on Clinical Outcomes in Haemodialysis Patients. Nephrol. Dial. Transplant..

[87] Miller Iii E.R., Pastor-Barriuso R., Dalal D., Riemersma R.A., Appel L.J., Guallar E. (2005). Meta-Analysis: High-Dosage Vitamin E Supplementation May Increase All-Cause Mortality Background: Experimental Models and Observational Studies. Ann. Intern. Med..

[88] Lonn E., Bosch J., Yusuf S., Sheridan P., Pogue J., Arnold J.M., Ross C., Arnold A., Sleight P., Probstfield J. (2005). Effects of Long-Term Vitamin E Supplementation on Cardiovascular Events and Cancer A Randomized Controlled Trial. JAMA.

[89] Galli F., Buoncristiani U., Conte C., Aisa C., Floridi A. (2004). Vitamin E in Uremia and Dialysis Patients. Ann. N. Y. Acad. Sci..

[90] Nanayakkara P.W.B., Kiefte-de Jong J.C., ter Wee P.M., Stehouwer C.D.A., van Ittersum F.J., Olthof M.R., Teerlink T., Twisk J.W.R., van Guldener C., Smulders Y.M. (2009). Randomized Placebo-Controlled Trial Assessing a Treatment Strategy Consisting of Pravastatin, Vitamin E, and Homocysteine Lowering on Plasma Asymmetric Dimethylarginine Concentration in Mild to Moderate CKD. Am. J. Kidney Dis..

[91] Mann J.F.E., Lonn E.M., Yi Q., Gerstein H.C., Hoogwerf B.J., Pogue J., Bosch J., Dagenais G.R., Yusuf S. (2004). Effects of Vitamin E on Cardiovascular Outcomes in People with Mild-to-Moderate Renal Insufficiency: Results of the HOPE Study. Kidney Int..

[92] Mann J.F.E., Sheridan P., McQueen M.J., Held C., Arnold J.M.O., Fodor G., Yusuf S., Lonn E.M. (2008). Homocysteine Lowering with Folic Acid and B Vitamins in People with Chronic Kidney Disease—Results of the Renal Hope-2 Study. Nephrol. Dial. Transplant..

[93] Brandenburg V.M., Reinartz S., Kaesler N., Krüger T., Dirrichs T., Kramann R., Peeters F., Floege J., Keszei A., Marx N. (2017). Slower Progress of Aortic Valve Calcification with Vitamin K Supplementation: Results from a Prospective Interventional Proof-of-Concept Study. Circulation.

[94] Elliott M.J., Booth S.L., Hopman W.M., Holden R.M. (2014). Assessment of Potential Biomarkers of Subclinical Vitamin K Deficiency in Patients with End-Stage Kidney Disease. Can. J. Kidney Health Dis..

[95] Grzejszczak P., Kurnatowska I. (2021). Role of Vitamin K in CKD: Is Its Supplementation Advisable in CKD Patients?. Kidney Blood Press. Res..

[96] Fulton R.L., McMurdo M.E., Hill A., Abboud R.J., Arnold G., Struthers A., Khan F., Vermeer C., Knapen M.H., Drummen N.E. (2016). Effect of Vitamin K on Vascular Health and Physical Function in Older People with Vascular Disease—A Randomised Controlled Trial. J. Nutr. Health Aging..

[97] Holden R.M., Morton A.R., Garland J.S., Pavlov A., Day A.G., Booth S.L. (2010). Vitamins K and D Status in Stages 3-5 Chronic Kidney Disease. Clin. J. Am. Soc. Nephrol..

[98] Westenfeld R., Krueger T., Schlieper G., Cranenburg E.C.M., Magdeleyns E.J., Heidenreich S., Holzmann S., Vermeer C., Jahnen-Dechent W., Ketteler M. (2012). Effect of Vitamin K 2 Supplementation on Functional Vitamin K Deficiency in Hemodialysis Patients: A Randomized Trial. Am. J. Kidney Dis..

